# Dual Function of a *in vivo* Albumin-Labeling Tracer for Assessment of Blood Perfusion and Vascular Permeability in Peripheral Arterial Disease by PET

**DOI:** 10.3389/fcvm.2022.738076

**Published:** 2022-02-08

**Authors:** Zhongchan Sun, Guang Tong, Yuanhui Liu, Hualin Fan, Weibin He, Bo Wang, Shuang Xia, Pengcheng He

**Affiliations:** ^1^Guangdong Provincial Key Laboratory of Coronary Heart Disease Prevention, Department of Cardiology, Guangdong Cardiovascular Institute, Guangdong Provincial People's Hospital, Guangdong Academy of Medical Sciences, Guangzhou, China; ^2^Department of Cardiology, Ganzhou Municipal Hospital, Ganzhou, China; ^3^Guangdong Provincial Key Laboratory of South China Structural Heart Disease, Department of Cardiac Surgery, Guangdong Cardiovascular Institute, Guangdong General Hospital, Guangdong Academy of Medical Sciences, Guangzhou, China; ^4^Department of Cardiac Surgery, Ganzhou Municipal Hospital, Ganzhou, China; ^5^School of Medicine, Guangdong Provincial People's Hospital, South China University of Technology, Guangzhou, China

**Keywords:** vascular permeability, PET, Evans blue, blood perfusion, hindlimb ischemia

## Abstract

**Background:**

Peripheral arterial disease (PAD) leads to tissue ischemia in the extremities. Enhanced vascular permeability plays a critical role in targeted delivery of drugs for effective therapeutic angiogenesis and resultant blood perfusion recovery. However, optimal tracers for evaluating this process in PAD patients are lacking. At this time, we employed a novel *in vivo* albumin-labeling tracer of dual function, termed as ^18^F-NEB, to assess blood perfusion as well as vascular permeability by positron emission tomography (PET).

**Methods and Results:**

After successful establishment of mouse hindlimb ischemia (HI) model, static PET imaging was performed 15 min and 2 h post injection (p.i.) of ^18^F-NEB at 1, 3, 5, 7, 10 and 14 days post-surgery respectively. Gradual recovery of blood supply was detected by PET scan 15 min p.i. and collaborated by serial Laser Doppler. In addition, the highest vascular permeability observed by high local uptake of ^18^F-NEB at 2 h p.i. was consistent with histological examinations. Furthermore, we quantitatively evaluated the effect of vascular endothelial growth factor (VEGF) stimulus on vascular permeability and blood perfusion by PET scan using ^18^F-NEB probe in HI model, which were also confirmed by immunohistological results.

**Conclusion:**

The application of ^18^F-NEB probe alone by PET can successfully achieve dual imaging of blood perfusion as well as vascular permeability at different time points p.i. and monitor their responses to therapy in PAD model. The simple labeling approach and multipurpose feature suggest the great promise of using this imaging probe in theranostic applications for treating ischemic disease.

## Introduction

As one of the most common vascular diseases, peripheral vascular disease (PAD) is prevalent in the elderly around the world (1). The impairment of blood flow to the tissues in PAD often arises from arterial stenosis or occlusion in the extremities, clinically manifested as slower healing, intermittent claudication, ulceration, gangrene and even non-traumatic amputation.

Current treatments for PAD aim to improve blood perfusion and include mechanical revascularization therapies and anti-platelet medications (2). However, either surgical intervention or stent implantation only targets the macrovascular system. To ameliorate the circulation through the microvascular system, proangiogenic growth factor treatment has been evaluated and demonstrated efficacy in preclinical studies (3, 4). Accumulation of growth factors to ischemic tissues is vital for therapeutic angiogenesis, which mainly depends on the vascular permeability (5, 6). Therefore, assessment of vascular permeability within ischemic limbs is of great value in angiogenesis monitoring, predicting drug delivery efficiency during proangiogenic therapy.

Reliable non-invasive imaging techniques for vascular permeability and angiogenesis assessment in PAD patients remains scarce. Doppler ultrasound imaging is time-consuming and can be easily disturbed by calcified lesion and of low accuracy (7). Optical imaging lacks the sensitivity and penetration depth needed for accurate quantification (8). PET shows great potential as a sensitive imaging tool with high spatial resolution and is able to provide information regarding multiple physiological and pathological aspects aided by novel molecular probes (9, 10).

Albumin is the most abundant plasma protein and thereby able to serve as a promising candidate for imaging probes and a carrier for therapeutic agents. Evans blue (EB), an azo dye that has high affinity with albumin (11), can permeate to the interstitial space along with albumin in tissues with increased vascular permeability. In preclinical studies, a measure of EB in extracted tissues is the gold standard to assess vascular permeability after intravenous injection of this dye (12). So far, EB can still serve as a surrogate marker for albumin and act as a sensitive marker of vascular leakage in a variety of inflamed or injured tissues (13).

In our previous studies, we have synthesized a new nuclide probe derived from EB, ^18^F-AIF-NOTA-EB (^18^F-NEB), for *in vivo* labeling of albumin (14). Due to simple synthesis and satisfying imaging quality, ^18^F-NEB has been used for multiple purposes *in vivo* imaging, e.g., blood-pool imaging (14) to assess cardiac function and vascular permeability evaluation in tumor (15). In the present study, we utilized this *in vivo* albumin-labeling tracer ^18^F-NEB to non-invasively monitor blood perfusion in the early phase post injection (p.i.) as well as the leakage of albumin from the vessel lumen in the late phase p.i. in a mouse PAD model.

## Materials and Methods

### Animals

All animal procedures were performed in accordance with the National Institutes of Health Guidelines for the Care and Use of Laboratory Animals and were approved by the Animal Ethics Committee of Guangdong Academy of Medical Sciences. FVB male mice were acquired from Harlan Laboratories Inc. (Frederick, MD), weighing 25–30 g and aging 6–7 weeks. All surgical processes and imaging scan session were performed under anesthesia with 1.0–2.0% isoflurane in oxygen delivered at a flow of 1.0 L/min.

### Hindlimb Ischemic Murine Model

An electric shaver was used to remove the hair on both hindlimbs of each mouse with the help of depilatory cream. The skin on the top of thighs was then incised to expose the arteries, veins, and nerves. The mouse hindlimbs mainly include the thigh muscles in the top layer and the gastrocnemius in the deep layer. For the induction of right hindlimb ischemia, we ligated and excised a section of femoral artery and all branches derived from this section of femoral artery. The proximal and distal ends of the superficial femoral artery and vein, as well as the origins of all branches were ligated with surgical silk, size 6-0. Each of the vessels surrounded by three ligated points was excised. Femoral nerve was carefully separated from the contaminant vessels and preserved. The sham operated left hindlimb served as a control with an incision in the skin of the thigh (16).

As stimulus for ischemic hindlimb, a single dose of VEGF (0.5 μg) in 100 μl PBS was injected into inflamed gastrocnemius muscles below the site of occlusion at three different sites at day 3 post surgery and daily thereafter for a total of 3 days The same amount of PBS (100 μl) alone was injected intramuscularly in the same way after the induction of ischemia, which served as the non-treatment group.

### Serial Laser Doppler Perfusion Imaging of Hindlimbs

Laser Doppler imaging (LDI) was used to serially monitor the blood flow of the ischemic hindlimbs. Briefly, anesthetized mice were placed in supine position on a 37.4–38.0°C heating pad and then imaged using an analyzer (PeriScan-PIM3 Perimed AB, Sweden). The blood perfusion was quantitatively evaluated by perfusion ratio (ratio of average LDI index of ischemic to non-ischemic hindlimb) by LDPI win 3.1.3 (Perimed AB,Sweden).

### Power Doppler and Color Doppler Scans of Hindlimbs

Before and 1 day post-surgery, Power Doppler Imaging (PDI) and Color Doppler Imaging (CDI) were performed using a Vevo 2100 Imaging Systems (VisualSonics, Inc., Toronto, ON, Canada). After FVB mice were anesthetized, flow velocity and spatial vascular profile in right hindlimb were tested by Power Doppler with a linear transducer in three-dimensional mode. Meantime, Color Doppler mode scan in three-dimension was also performed to provide a visual overview of flow within the normal and ischemic skeletal muscle tissue as well as delineate the flow direction and velocity by red and blue color spectra.

### Preparation of ^18^F-AlF-NOTA-EB

The synthesis of ^18^F-NEB was performed according to the procedure reported previously (14). In brief, 0.13 ml of acetonitrile and 0.05 ml of aqueous ^18^F-fluoride (0.3–0.9 GBq) were added to a 1-ml plastic tube containing 3 ml of 2 mM aluminum chloride in 0.5 M, pH 4.0, sodium acetate buffer and 6 ml of 3 mM NEB in 0.5 M, pH 4.0, sodium acetate buffer. The mixture was stirred in a vortex mixer and heated in a 105°C heating block for 10 min. The vial was cooled, and the solution was diluted with 10 ml of water and trapped on a Varian Bond Elut C_18_ column (100 mg). The radioactivity trapped on the C_18_ column was eluted with 0.3 ml of 80% ethanol/water containing 1 mM HCl. The ethanol solution was evaporated with argon flow, and the final product was dissolved in phosphate-buffered saline (PBS) and analyzed by HPLC (15).

### Small Animal PET Imaging

PET scans were performed using an INVEON small animal PET scanner (Siemens Preclinical Solutions). As designed, PET scan was done at 1, 3, 5, 7, 10, and 14 days post-surgery respectively. At each day mentioned, mice were anesthetized and approximately 3.75 MBq (100 μCi) of ^18^F-NEB tracer was administered *via* tail vein injection. Then 10-min static PET images were acquired at 15 min and 2 h after tracer injection respectively. The images were reconstructed by a three-dimensional ordered subsets expectation maximization (3D-OSEM) algorithm and analyzed using ASI Pro VMTM software. The mean (%ID) uptake of the ischemic hindlimb tissue was determined by drawing three-dimensional regions of interest (ROIs) surrounding an entire limb on the coronal images. The radioactivity contained in the ROI divided by the dose administered to the animal gave the %ID. Each group contained 5–6 mice.

### Immunohistochemistry Assay

Ischemic skeletomuscular tissues at different time points post HI surgery were harvested and fixed in 4% paraformaldehyde solution, dehydrated through graded solutions of ethanol, and embedded in paraffin. Serial sections (5 μm thick) were cut and mounted on glass slides (Fisher, Pittsburgh, PA). After dewaxing and microwave antigen retrieval, slides were incubated with 10% normal goat serum for 1 h and then overnight at 4°C with mouse monoclonal antibody to CD31 (1:100) or PBS as a control. Biotinylated anti-mouse IgG (Sigma) was applied and detected with a streptavidin-peroxidase complex and 0.1% of 3,3′-diaminobenzidine (Sigma) in PBS with 0.05% H_2_O_2_ for 5 min at room temperature. In addition, slides were stained with hematoxylin and eosin (H & E) for tissue morphology (17).

### Statistical Analysis

Results were expressed as mean and standard deviation (mean ± SD). Statistical analysis was performed using one-way ANOVA followed by the Bonferroni multiple comparison test or Student *t*-test. *P* < 0.05 was considered as statistically significant.

## Results

### Mouse Hindlimb Ischemia Model

**Figure 1:**
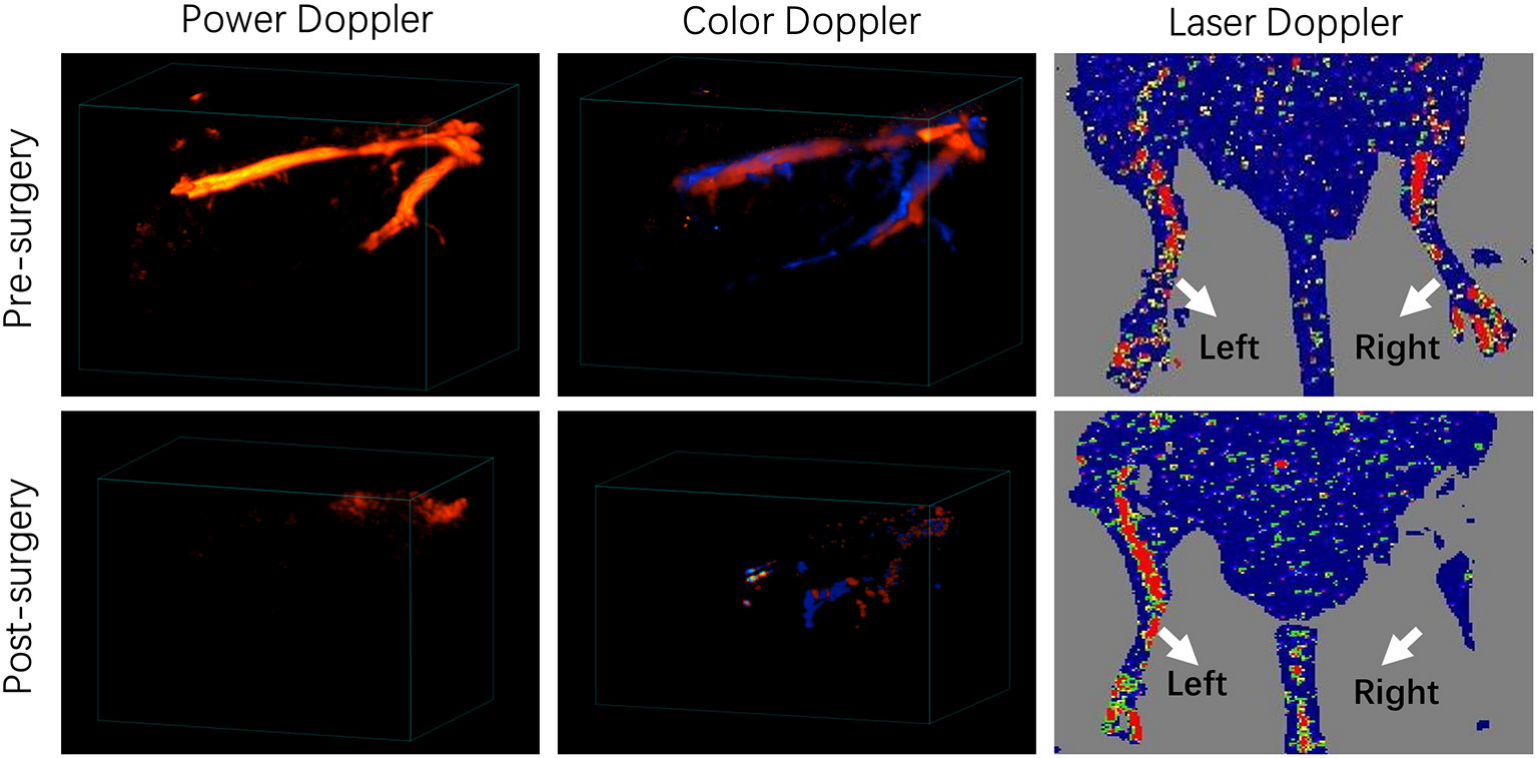
Characterization of a murine hindlimb ischemia (HI) model. Three-dimensional power Doppler, color Doppler and Laser Doppler imaging of pre-surgery and post-surgery hindlimbs. Red and blue colors obtained by Color Doppler imaging represents blood flow with different directions, which flows toward and away from the transducer, respectively.

We used a well-established mouse hindlimb ischemia (HI) model to mimic PAD. Ligation and excision of a section of femoral artery and all branches originated from this section were performed in the right hindlimb of FVB mice. The sham operated left hindlimb served as the control. Laser Doppler Imaging (LDI) showed significantly diminished blood flow in the right hindlimb at day 1 post surgery (Figure 1). Power Doppler Imaging (PDI) (18) and Color Doppler Imaging (CDI) were also utilized to assess the blood perfusion within ischemic area. Both three-dimensional PDI and CDI scans displayed the abrupt blockade of blood flow and the absence of multiple branches in femoral artery at the site of occlusion, as compared to the clear and intact vasculature before the surgery (Figure 1). All these results indicated successful establishment of mouse hindlimb ischemia model.

### The Principle of ^18^F-NEB Application in HI Model

**Figure 2:**
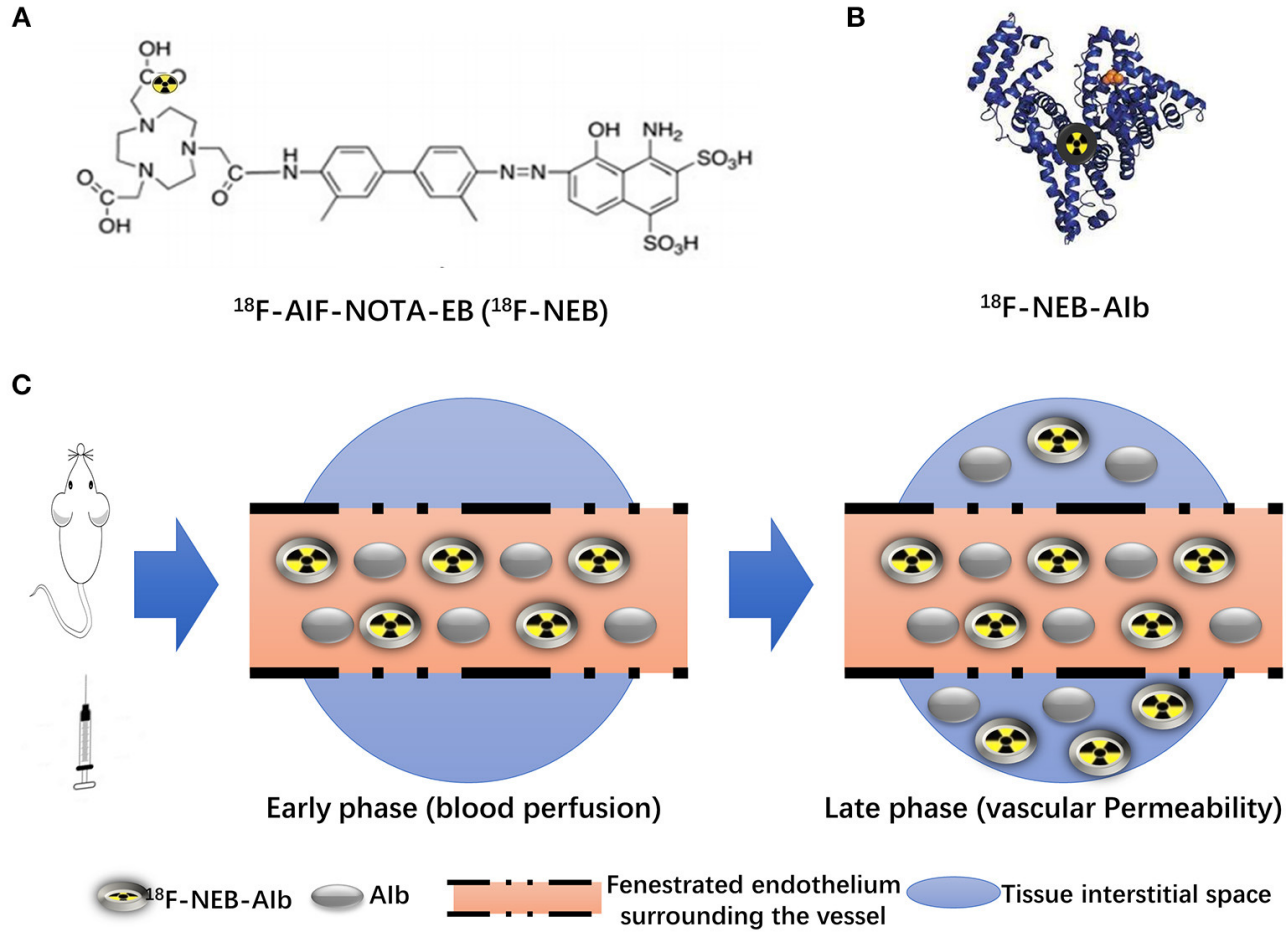
Principle of ^18^F-NEB application in HI model. **(A)** Chemical structure of NOTA conjugated truncated Evan's Blue (NEB) and radiolabeling of NEB with ^18^F-aluminum (^18^F-AIF-NOTA-EB/^18^F-NEB). **(B)** The binding of ^18^F-AIF-NOTA-EB with serum albumin, forming the macromolecule^18^F-AIF-NOTA-EB-AIb (^18^F-NEB-AIb). **(C)** Schematic diagram of ^18^F-NEB imaging. After i.v injection of ^18^F-NEB into surgical mice, sectional serum albumin was labeled with the probe immediately. In the early phase, circulation of ^18^F-NEB-AIbwithin the vessel lumen enables the blooding pool imaging as well as the blood flow perfusion imaging in the hindlimb region. Then the ^18^F-NEB-AIb gradually extravagated through the fenestrated endothelial junctions into to interstitial space, allowing for vascular permeability evaluation in the late phase.

As the chemical structure in Figure 2A showed, ^18^F-AIF-NOTA-EB (^18^F-NEB) was developed by labeling a NOTA (1,4,7-triazacyclononane-N,N′,N″-triaceticacid)- conjugated truncated EB (NEB) with ^18^F through the formation of ^18^F-aluminum- fluoride complex. Due to the high concentration of albumin in the blood circulation, only a trace amount of NEB is needed for NEB-albumin complex formation and equilibrium, the process of which takes just a few minutes (Figure 2B). In the early phase post injection (p.i.), most of the ^18^F-NEB-albumin complex is restrained in the blood circulation (Supplementary Figure S1), allowing assessment of blood volume (Figure 2C). In response to inadequate blood flow and oxygenation, acute inflammation is initiated and characterized by significant immunovascular changes (19), involving vasodilation, increased vascular permeability and the movement of plasma fluid containing plenty of serum proteins (20). Gradually, ^18^F-NEB-albumin complex is extravasated into interstitial space. Therefore, PET imaging with radiolabeled NEB in the late phase p.i. permits non-invasive evaluation of vascular permeability in HI model (Figure 2C).

### PET Imaging of Blood Perfusion

**Figure 3:**
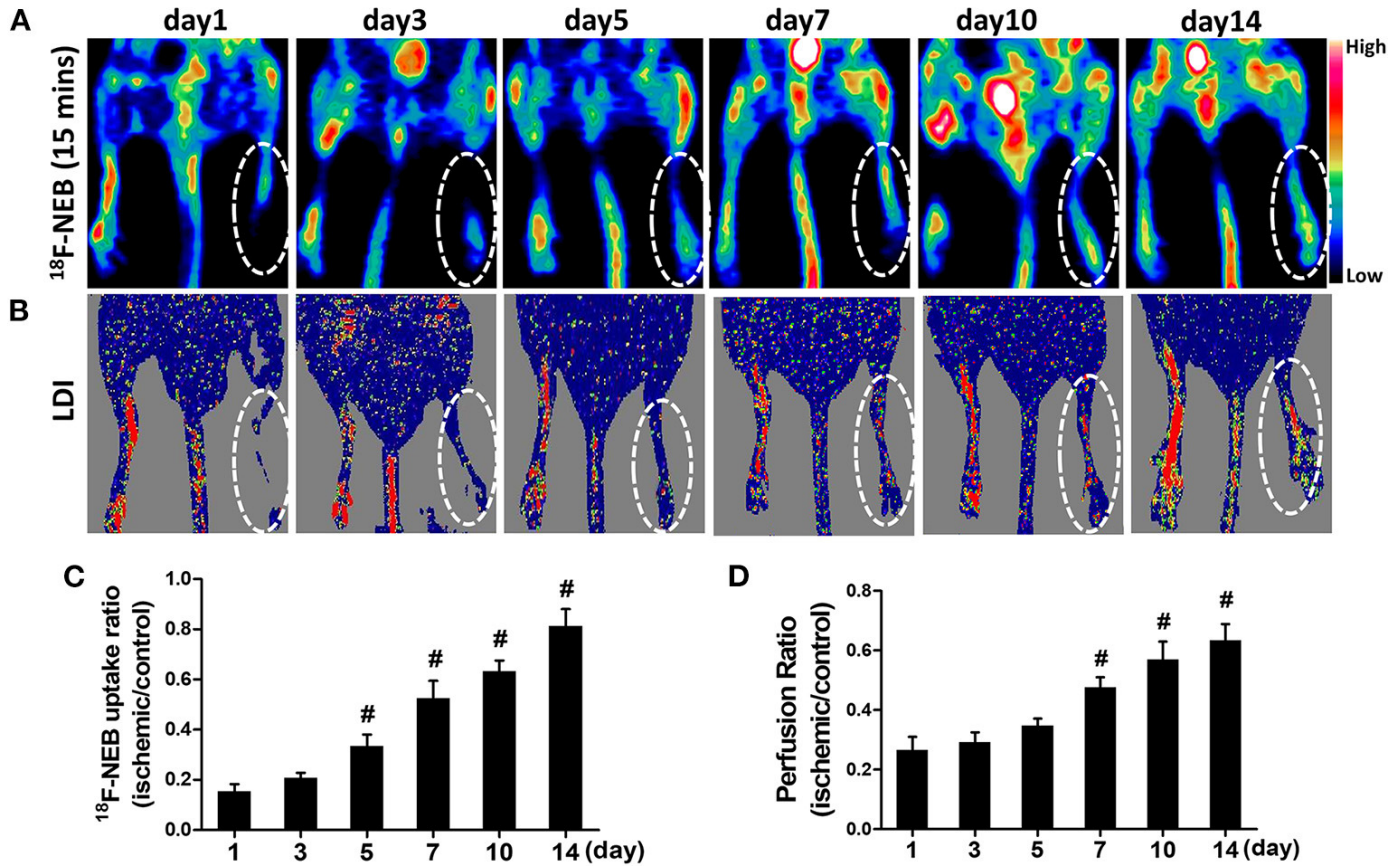
Gradual recovery of blood perfusion was detected by PET imaging at early time (15 min) post ^18^F-NEB injection **(A)**, indicating angiogenesis. The similar tendency was also confirmed by serial Laser Doppler imaging **(B)**. PET imaging and serial Laser Doppler imaging were performed at day 1, day 3, day 5, day 7, day 10 and day 14 post surgery, respectively. Analysis of the ^18^F-NEB uptake ratio (ischemic/control hindlimb) by PET **(C)** and the perfusion ratio (ischemic/control hindlimb) by serial LDI **(D)** at a serial of time points (^**#**^*P* < 0.05, vs. day 1).

Fifteen minutes after intravenous administration of ^18^F-NEB probe (100 μCi per mouse), static PET scans were performed to assess blood perfusion at 1, 3, 5, 7, 10, and 14 days post-surgery, respectively. As shown in Figure 3A, ^18^F-NEB signal intensity in surgical hindlimb (area within white dotted line) was significantly diminished in the first day post-surgery (0.154±0.029) and gradually increased over time, while the non-surgical hindlimb showed no changes. At 14 days post-surgery (0.812 ± 0.068), local signal of the surgical hindlimb was significantly augmented, suggesting recovery of blood perfusion within ischemic muscle. Serial Laser Doppler imaging (LDI) was also performed to monitor the changes of blood flow, which was in consistent with the PET images (Figure 3B).

Both the quantification data of PET and LDI demonstrated the similar trend of perfusion recovery in ischemic hindlimb. There has been significant increase of blood perfusion in ischemic hindlimb since day 5 post surgery (0.334 ± 0.047) based on PET data (Figure 3C). However, LDI did not recognize this remarkable change until day 7 post surgery (0.476±0.034) (Figure 3D), which may be due to the relatively low sensitivity of LDI.

### PET Imaging of Vascular Permeability

**Figure 4:**
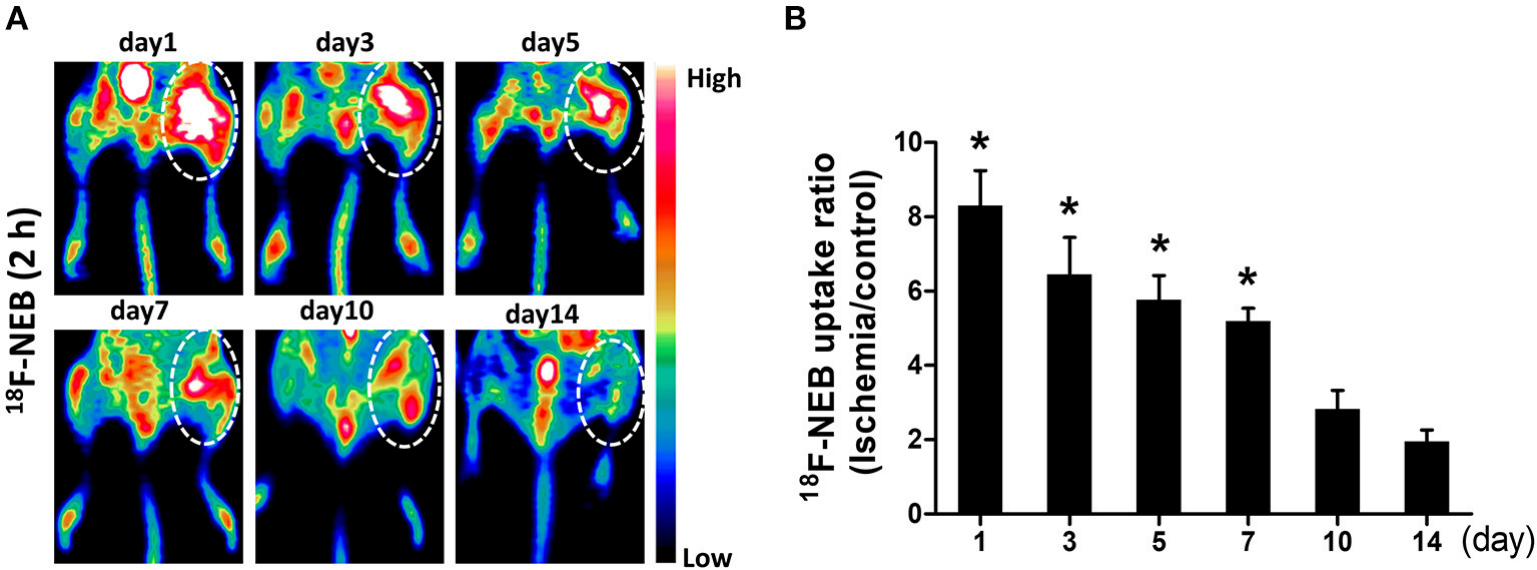
Vascular permeability in ischemic limb was monitored by PET imaging. **(A)** representative coronal images of PET scan 2 h after ^18^F-NEB tracer injection at day 1, day 3, day 5, day 7, day 10 and day 14 post surgery, respectively. **(B)** Analysis of PET data to determine the uptake ratio of ^18^F-NEB tracer within ischemic right limb and control left limb at a serial of time points (^*^P < 0.05, vs. day 14).

Following the first PET scan at 15 min post ^18^F-NEB injection, the same batch of surgical mice were further scanned at 2 h p.i. to monitor the inflammatory leakage of albumin from the vessel within the ischemic region of occlusive hindlimb. Figure 4A showed the representative coronal PET images of mice hindlimbs at multiple designated time points (day 1, 3, 5, 7, 10, and 14) post-surgery. According to the PET imaging results, we observed the highest radioactivity level of ^18^F-NEB probe within ischemic right hindlimb in the first day (8.303±2.1), indicating the increased vascular permeability during the acute inflammation phase. Then, lessened leakage of serum albumin was revealed by the decreased radioactivity within 2 weeks (day 14: 1.953 ± 0.69). The probe uptake within ischemic right limb and non-ischemic left limb was quantified based on PET images and presented as Figure 4B. Based on the quantitative data, the high uptake ratio (ischemic limb to non-ischemic) of ^18^F-NEB was maintained until day 7 post surgery.

### Histology Analysis of Ischemic Hindlimbs

**Figure 5:**
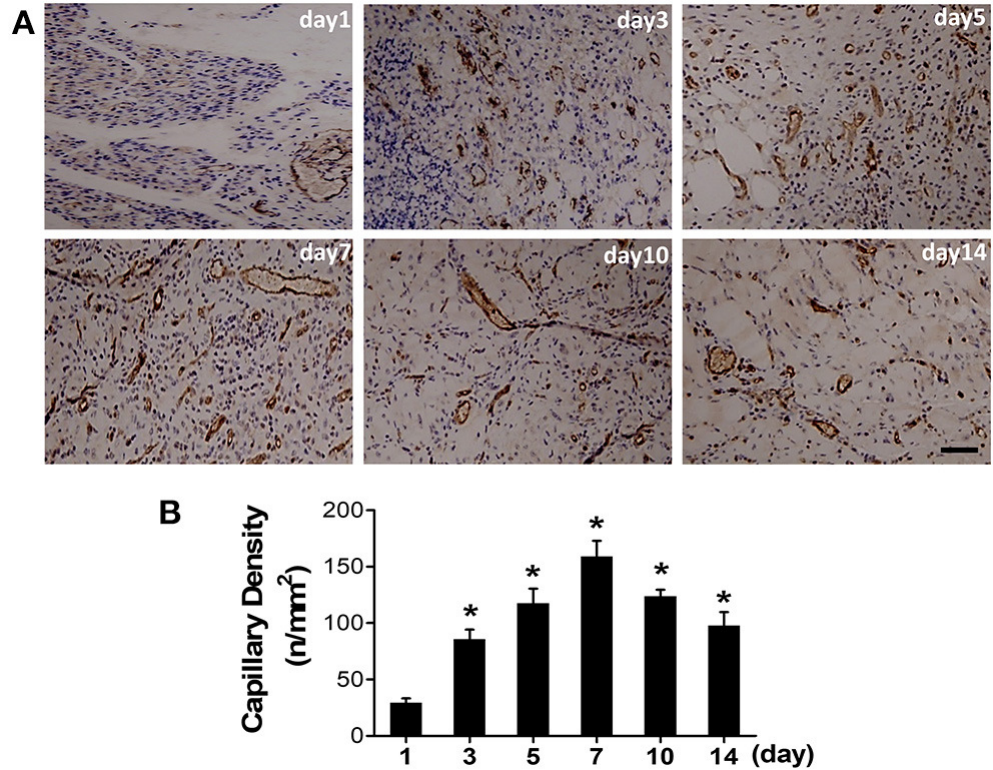
Angiogenesis progression after the induction of ischemia. **(A)** Representative photographs of CD31 positive vessel were immunohistochemically detected at a serial of time points after surgery. **(B)** Quantitative analysis of CD31 capillary density in each group (^*^P < 0.05, vs. day 1).

Ischemic skeletomuscular of surgical mice were also collected for histology analysis. Immunohistochemical (IHC) staining with anti-CD31 (marker for endothelial cell) antibody were performed (Figure 5A). IHC staining results showed the irregular vessel lumen (highlighted by brown color), tangled endothelial cells arrangement and swelling interstitial space at the first day post-surgery, compared with the pre-surgery one (Supplementary Figure S2). Meanwhile, vast neutrophils (nucleus in blue or purple) surrounding the damaged lumen indicated the initial leakage of plasma fluid from the vessel. The irregular vessel lumen and intense neutrophil infiltration in the ischemic skeleton-muscular tissue reflected initial enhanced vascular permeability at the acute stage. From day 5 (117.6 ± 12.93), newly regenerated capillaries were prevalent and reached the peak at day 7 (159.0 ± 13.95), as displayed by CD31 staining (Figure 5B).

### Monitoring the Alteration of Blood Perfusion and Vascular Permeability in Response to VEGF Treatment Using ^18^F-NEB

**Figure 6:**
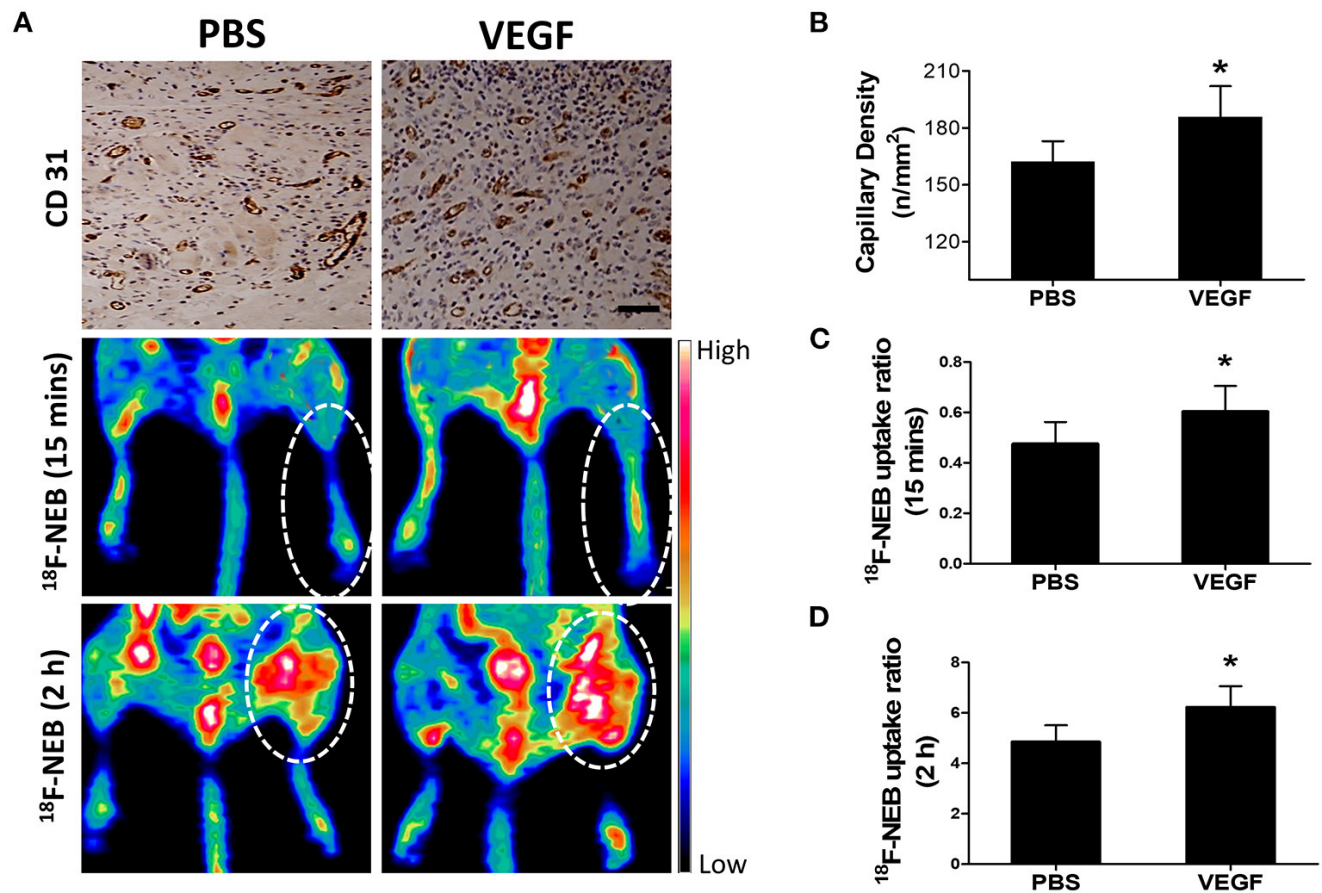
Effect of VEGF-induced angiogenesis on vascular permeability in ischemic limb at day 7 post-operation. **(A)** Immunohistochemical staining of CD 31 (panel left) to detect angiogenesis in the ischemic skeletomuscular tissue sections; representative PET images of ischemic hindlimb at early phase (15 min: middle panel) and late phase (2 h: right panel) after ^18^F-NEB tracer injection in the VEGF-treated (VEGF) and PBS-treatment (PBS) groups. **(B)** Quantitative analysis of CD31 capillary density at day 7 post-operation in mice treated with VEGF or PBS (*P < 0.05, vs. PBS; *P* = 0.015). **(C,D)** Quantification of the signal intensity from *in vivo* PET imaging in early phase **(C)** (*P* = 0.027) and late phase **(D)** (*P* = 0.005) after ^18^F-NEB tracer injection in mice treated with VEGF or PBS (*P < 0.05 vs. PBS).

Given the fact that angiogenesis development in HI can lead to both blood perfusion and vascular permeability enhancement, we next used vascular endothelial growth factor (VEGF), the most widely studied angiogenic stimuli, to treat ischemic limbs in our study. Based on previous reports and our histological data as shown in Figure 5, we chose day 7 post surgery, the peak time of angiogenesis, to assess the response to VEGF treatment. As expected, a significant increase of the capillary number at 7 days post-surgery was evidenced by CD31 staining in VEGF-treated group (Figure 6A: upper row). Quantitative data showed the capillary density of 185.7±16.27 at VEGF-treated group and 162.3 ± 10.65 at PBS-treated group (Figure 6B).

Next, we evaluated whether ^18^F-NEB was capable of assessing the alteration of blood perfusion and vascular permeability in response to VEGF treatment in HI model. Figure 6A showed representative coronal PET images of hindlimbs at 15 min (Figure 6A: middle row) and 2 h (Figure 6A: lower row) p.i, of ^18^F-NEB, respectively. Based on PET quantification, the ^18^F-NEB uptake ratio of ischemic hindlimb to control one in the early phase (Figure 6C) in VEGF-treated group (0.6 ± 0.1) was significantly higher than PBS-treated group (0.48 ± 0.087; *P* < 0.05). In the late phase (Figure 6D), the ^18^F-NEB uptake ratio was similarly elevated by VEGF treatment (6.23 ± 0.83) as compared to PBS treatment (4.85 ± 0.66; *P* < 0.05). These imaging results were consistent with CD31 staining. All these data demonstrated that the resulting blood perfusion and vascular permeability enhancement of VGEF-induced angiogenesis in ischemic tissues could be non-invasively detected by ^18^F-NEB probe.

## Discussion

Peripheral arterial disease (PAD) has become a global health concern and is the leading cause of non-injury amputation in the elderly. Tissue ischemia-induced vascular permeability enhancement and active angiogenesis interact with each other, which plays a critical role in blood perfusion recovery. In the present study, we evaluated the potential of utilizing a dual function tracer ^18^F-NEB of convenience and safety to dynamically and non-invasively monitor the blood perfusion as well as the vascular permeability in a mouse hindlimb ischemia (HI) model.

Immediately after intravenous injection of ^18^F-NEB, most of the radioactivity was retained in the circulatory system as expected. Our previous study has successfully utilized ^18^F-NEB to evaluate cardiac function under both physiologic and pathologic conditions. Compared to conventional *in vitro* labeling of albumin through ^18^F-FB-albumin (21), *in vivo* labeling of albumin with ^18^F-NEB is advantageous. The preparation procedure of *in vivo* labeled albumin is rapid and efficient through a newly developed truncated EB derivative. Meanwhile, *in vitro* radiolabeling autologous blood products impose high risks on all contacts, including operators and patients. In addition, due to the abundant albumin in the blood circulation and the high affinity of EB dye with albumin, quite small amount of radiolabeled NEB is needed for imaging, which guarantees the safe practice (22). Thus, this novel PET tracer has great potential for clinical translation.

The high resolution and quantitative feature of PET also ensure the accuracy of evaluation. As compare with PET imaging, LDI is time-consuming and can be interfered by environmental temperature. In addition, PET imaging is able to penetrate into deep tissue and construct three-dimensional (3D) images (Supplementary Video S1), resulting in more accurate evaluation. In the present study, PET imaging could recognize the significant improvement of blood flow perfusion as early as day 5, as compared to LDI detection at day 7. Apart from blood perfusion assessment, ^18^F-NEB can also be applied to evaluate vascular permeability in tumors, inflammatory diseases and ischemic tissues. In our previous study, both turpentine-induced acute inflammation and xenografted tumor models were established to evaluate increased vascular permeability by using ^18^F-NEB (15). Herein, we took advantages of these two functions of ^18^F-NEB in one animal model to non-invasively visualize the circulatory distribution as well as local accumulation of serum albumin by PET.

Earlier reports suggests that the acute inflammation induced by femoral artery occlusion, normally consists of a vascular phase followed by a cellular phase (23). Initially, inflammation is characterized by vascular dilation and enhanced permeability, which in turn lead to the leakage of plasma fluid. Indeed, we observed highest uptake of ^18^F-NEB at day 1, indicating significantly enhanced vascular permeability at the acute stage. However, the high uptake ratio (ischemic limb to non-ischemic) of ^18^F-NEB was maintained until day 7 post surgery, which cannot be simply explained by acute vasodilation and resultant enhanced leakage of albumin from vessel lumen. In fact, in the late phase, upregulated angiogenic factors in ischemic tissue may activate and mobilize endothelial cells to form new leaky vessels, termed as angiogenesis (24). Inflammation and angiogenesis are intertwined with each other (25, 26), leading to persistent accumulation of circulating radiolabeled tracers within ischemic limb post-surgery.

To evaluate whether ^18^F-NEB is capable of assessing blood perfusion and vascular permeability in response to treatment, VEGF was used to activate angiogenesis and form more leaky capillaries. Early time post intravenous injection, elevated ^18^F-NEB signal intensity in surgical hindlimb indicated improvement of blood reperfusion stimulated by VEGF, justifying the feasibility of ^18^F-NEB serving as a blood-pool imaging agent. Late time post intravenous injection, higher ^18^F-NEB uptake in ischemic tissue indicated vascular permeability enhancement, which is also closely related with VEGF-induced angiogenesis. Our data demonstrated that VEGF-induced enhancement of both blood perfusion and vascular permeability in ischemic tissues could be monitored by ^18^F-NEB alone. Real-time and non-invasive evaluation of the treatment response will provide detailed information to predict drug delivery efficiency and guide the individualized therapeutic strategy during the development and treatment of HI. It is worth to notice that even in delayed ^18^F-NEB PET imaging (2 h p.i.), the influence of blood perfusion exists. However, the tracer accumulation at this time is mainly resulted from albumin/NEB complex extravasation rather than that still in blood vessels. Thus, we believe delayed ^18^F-NEB PET dominantly reflect vascular permeability.

For clinical application, apart from PAD, there are also some other factors that can alter the permeability of ^18^F-NEB. Malignant tumors often show increased uptake and retention of high-molecular-weight non-targeted drugs and prodrugs, which is known as the enhanced permeability and retention (EPR) effect (27). EPR in tumors has been attributed to vascular permeability enhancement and decreased efflux of macromolecules from the pathological locus. Several features of infection-induced inflammation resemble these processes. Therefore, enhanced permeability of ^18^F-NEB can also occur in solid tumor, infective tissue or inflammatory lesion in patients.

In conclusion, benefiting from the high sensitivity of PET imaging and the ideal half-life of ^18^F, we took advantage of a *in vivo* albumin-labeling tracer ^18^F-NEB of dual function to dynamically and noninvasively monitor the blood perfusion in the early phase as well as the vascular permeability in the late phase p.i. in a mouse hindlimb ischemia model, which mirrored the evolution of inflammation and angiogenesis after femoral artery occlusion. Moreover, this single nuclide probe of safety and convenience could monitor the alteration of both blood perfusion and vascular permeability in response to therapy. The simple labeling approach and multipurpose feature suggest the great promise of using this imaging probe in theranostic applications for treating ischemic disease.

## Data Availability Statement

The raw data supporting the conclusions of this article will be made available by the authors, without undue reservation.

## Ethics Statement

The animal study was reviewed and approved by the Animal Ethics Committee of Guangdong Academy of Medical Sciences.

## Author Contributions

ZS, GT, and PH conceived and designed the study. ZS, GT, YL, HF, WH, BW, and SX performed major experiments. GT, YL, and SX collected and analyzed the data. GT, ZS, and YL wrote the manuscript. All authors contributed to and approved the manuscript.

## Funding

This study was supported by the Guangdong Provincial People's Hospital Cardiovascular Research Fund (2020XXG004), National Natural Science Foundation of Guangdong Provincial People's Hospital (KY012020289), and Guangdong Provincial People's Hospital Clinical Research Fund (Y012018085). None of these funding sources had any role in writing the manuscript or the decision to submit for publication.

## Conflict of Interest

The authors declare that the research was conducted in the absence of any commercial or financial relationships that could be construed as a potential conflict of interest.

## Publisher's Note

All claims expressed in this article are solely those of the authors and do not necessarily represent those of their affiliated organizations, or those of the publisher, the editors and the reviewers. Any product that may be evaluated in this article, or claim that may be made by its manufacturer, is not guaranteed or endorsed by the publisher.

## Supplementary Material

Supplementary Figure S1 PET imaging was performed 15 min post injection of ^18^F-NEB.

Supplementary Figure S2 Representative photograph of CD31 immunohistochemical staining before surgery.

Supplementary Figure S3 H & E stained images of major organs (heart, lung, liver and kidney), harvested from mice 24 h after injection with/without ^18^F-NEB.

Supplementary Video S1 PET images were reconstructed by a three-dimensional ordered subsets expectation maximization (3D-OSEM) algorithm.

## References

[1] Fowkes FG Aboyans V Fowkes FJ McDermott MM Sampson UK Criqui MH. Peripheral artery disease: epidemiology and global perspectives. Nat Rev Cardiol. (2017) 14:156–70. 10.1038/nrcardio.2016.179 27853158

[2] Morley RL Sharma A Horsch AD Hinchliffe RJ. Peripheral artery disease. BMJ. (2018) 360:j5842. 10.1136/bmj.j5842 29419394

[3] Amsden BG. Delivery approaches for angiogenic growth factors in the treatment of ischemic conditions. Expert Opin Drug Deliv. (2011) 8:873–90. 10.1517/17425247.2011.577412 21644842

[4] Sun Z Huang P Tong G Lin J Jin A Rong P. VEGF-loaded graphene oxide as theranostics for multi-modality imaging-monitored targeting therapeutic angiogenesis of ischemic muscle. Nanoscale. (2013) 5:6857–66. 10.1039/c3nr01573d 23770832 PMC4607062

[5] Dvorak HF Nagy JA Feng D Brown LF Dvorak AM. Vascular permeability factor/vascular endothelial growth factor and the significance of microvascular hyperpermeability in angiogenesis. Curr Top Microbiol Immunol. (1999) 237:97–132. 10.1007/978-3-642-59953-8_6 9893348

[6] Dvorak HF Brown LF Detmar M Dvorak AM. Vascular permeability factor/vascular endothelial growth factor, microvascular hyperpermeability, and angiogenesis. Am J Pathol. (1995) 146:1029–39. 7538264 PMC1869291

[7] Pollak AW Norton PT Kramer CM. Multimodality imaging of lower extremity peripheral arterial disease: current role and future directions. Circ Cardiovasc Imaging. (2012) 5:797–807. 10.1161/CIRCIMAGING.111.970814 23169982 PMC3504350

[8] Kim J Cao L Shvartsman D Silva EA Mooney DJ. Targeted delivery of nanoparticles to ischemic muscle for imaging and therapeutic angiogenesis. Nano Lett. (2011) 11:694–700. 10.1021/nl103812a 21192718 PMC3073422

[9] Allen-Auerbach M Weber WA. Measuring response with FDG-PET: methodological aspects. Oncologist. (2009) 14:369–77. 10.1634/theoncologist.2008-0119 19357228

[10] Gao H Lang L Guo N Cao F Quan Q Hu S. PET imaging of angiogenesis after myocardial infarction/reperfusion using a one-step labeled integrin-targeted tracer 18F-AlF-NOTA-PRGD2. Eur J Nucl Med Mol Imaging. (2012) 39:683–92. 10.1007/s00259-011-2052-1 22274731 PMC3319105

[11] Spahr PF Edsall JT. Amino acid composition of human and bovine serum mercaptalbumins. J Biol Chem. (1964) 239:850–4. 10.1016/S0021-9258(18)51668-9 14154465

[12] Gibson JG Evans WA. Clinical Studies of the Blood Volume. I Clinical Application of a Method Employing the Azo Dye “Evans Blue” and the Spectrophotometer. J Clin Invest. (1937) 16:301–16. 10.1172/JCI100859 16694480 PMC424872

[13] Moitra J Sammani S Garcia JG. Re-evaluation of Evans Blue dye as a marker of albumin clearance in murine models of acute lung injury. Transl Res. (2007) 150:253–65. 10.1016/j.trsl.2007.03.013 17900513

[14] Niu G Lang L Kiesewetter DO Ma Y Sun Z Guo N. *In Vivo* Labeling of Serum Albumin for PET. J Nucl Med. (2014) 55:1150–6. 10.2967/jnumed.114.139642 24842890 PMC4576845

[15] Chen H Tong X Lang L Jacobson O Yung BC Yang X. Quantification of tumor vascular permeability and blood volume by positron emission tomography. Theranostics. (2017) 7:2363–76. 10.7150/thno.19898 28744320 PMC5525742

[16] Fan W Han D Sun Z Ma S Gao L Chen J. Endothelial deletion of mTORC1 protects against hindlimb ischemia in diabetic mice *via* activation of autophagy, attenuation of oxidative stress and alleviation of inflammation. Free Radic Biol Med. (2017) 108:725–40. 10.1016/j.freeradbiomed.2017.05.001 28473248

[17] Sun Z Shen L Sun X Tong G Sun D Han T. Variation of NDRG2 and c-Myc expression in rat heart during the acute stage of ischemia/reperfusion injury. Histochem Cell Biol. (2011) 135:27–35. 10.1007/s00418-010-0776-9 21193923

[18] Sun Z Ma N Fan W Guo L Chen J Zhu L. Noninvasive monitoring of the development and treatment response of ischemic hindlimb by targeting matrix metalloproteinase-2 (MMP-2). Biomater Sci. (2019) 7:4036–45. 10.1039/C9BM00915A 31482934

[19] Krishna SM Moxon JV Golledge J. A review of the pathophysiology and potential biomarkers for peripheral artery disease. Int J Mol Sci. (2015) 16:11294–322. 10.3390/ijms160511294 25993296 PMC4463701

[20] Azzopardi EA Ferguson EL Thomas DW. The enhanced permeability retention effect: a new paradigm for drug targeting in infection. J Antimicrob Chemother. (2013) 68:257–74. 10.1093/jac/dks379 23054997

[21] Kilbourn MR Dence CS Welch MJ Mathias CJ. Fluorine-18 labeling of proteins. J Nucl Med. (1987) 28:462–70. 3494825

[22] Giger M Baumgartner HR Zbinden G. Toxicological effects of Evans blue and Congo red on blood platelets. Agents Actions. (1974) 4:173–80. 10.1007/BF01970259 4416679

[23] Brevetti G Giugliano G Brevetti L Hiatt WR. Inflammation in peripheral artery disease. Circulation. (2010) 122:1862–75. 10.1161/CIRCULATIONAHA.109.918417 21041698

[24] Krishna SM Omer SM Golledge J. Evaluation of the clinical relevance and limitations of current pre-clinical models of peripheral artery disease. Clin Sci. (2016) 130:127–50. 10.1042/CS20150435 26678170

[25] Guo L Akahori H Harari E Smith SL Polavarapu R Karmali V. CD163+ macrophages promote angiogenesis and vascular permeability accompanied by inflammation in atherosclerosis. J Clin Invest. (2018) 128:1106–24. 10.1172/JCI93025 29457790 PMC5824873

[26] Szade A Grochot-Przeczek A Florczyk U Jozkowicz A Dulak J. Cellular and molecular mechanisms of inflammation-induced angiogenesis. IUBMB Life. (2015) 67:145–59. 10.1002/iub.1358 25899846

[27] Maeda H. Tumor-selective delivery of macromolecular drugs *via* the EPR effect: background and future prospects. Bioconjug Chem. (2010) 21:797–802. 10.1021/bc100070g 20397686

