# Supplementary figures and images for: Dual Function of a in vivo Albumin-Labeling Tracer for Assessment of Blood Perfusion and Vascular Permeability in Peripheral Arterial Disease by PET
Source: Front Cardiovasc Med. 2022 Feb 8;9:738076. doi: 10.3389/fcvm.2022.738076 (PMC8860820; doi:10.3389/fcvm.2022.738076)

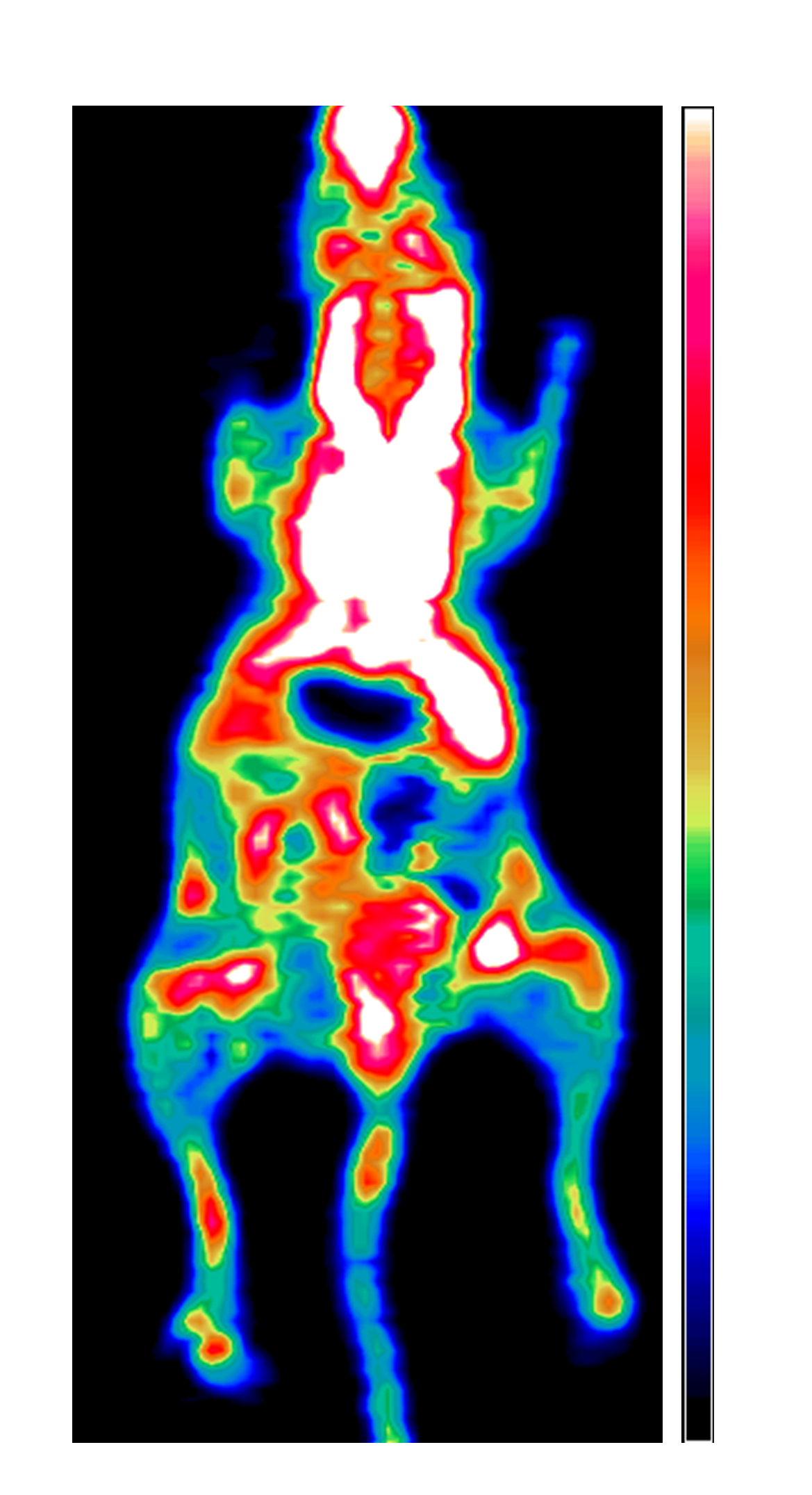

Supplement: Supplementary Figure S1 — PET imaging was performed 15 min post injection of 18F-NEB. [file Image_1.TIF]

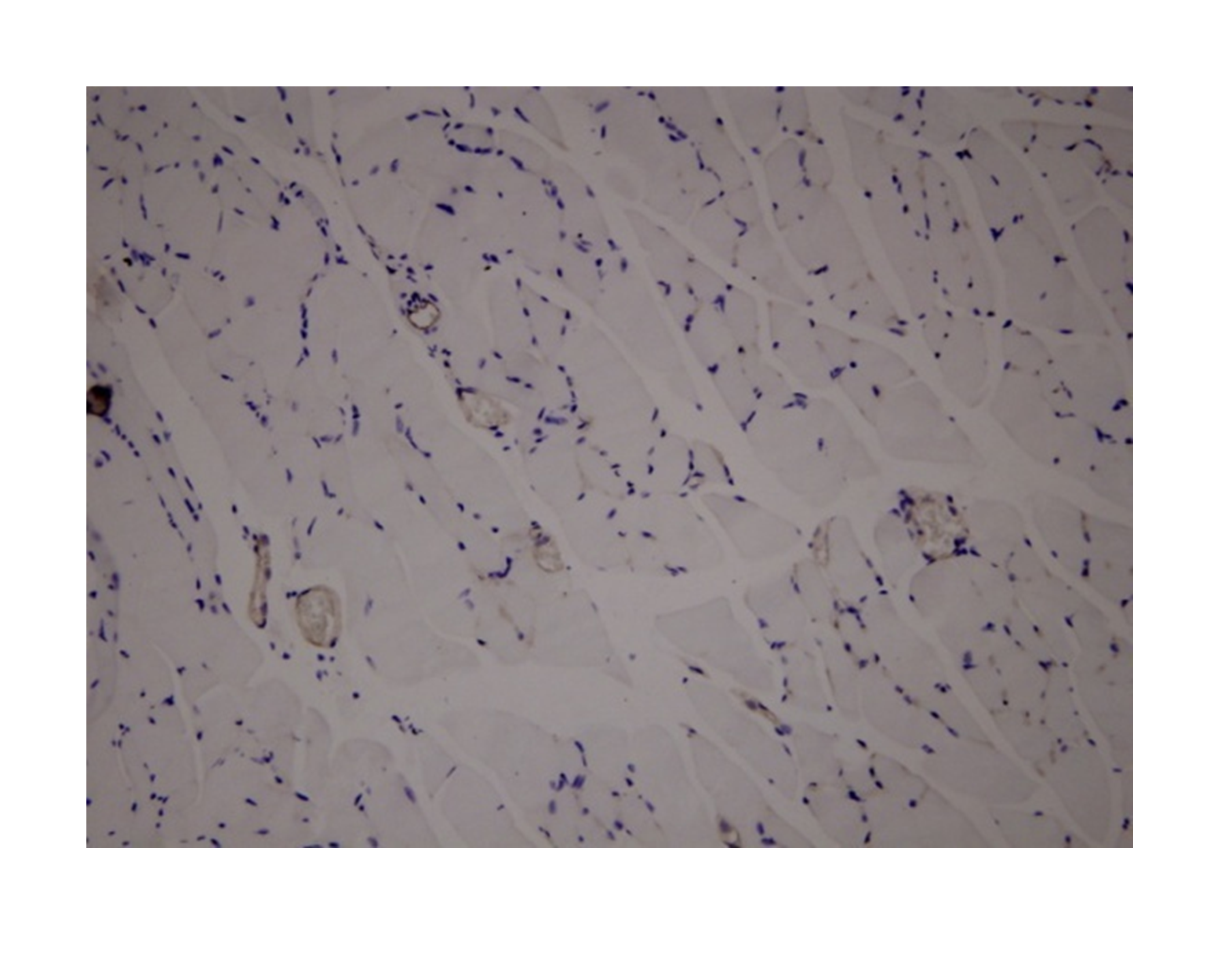

Supplement: Supplementary Figure S2 — Representative photograph of CD31 immunohistochemical staining before surgery. [file Image_2.TIF]

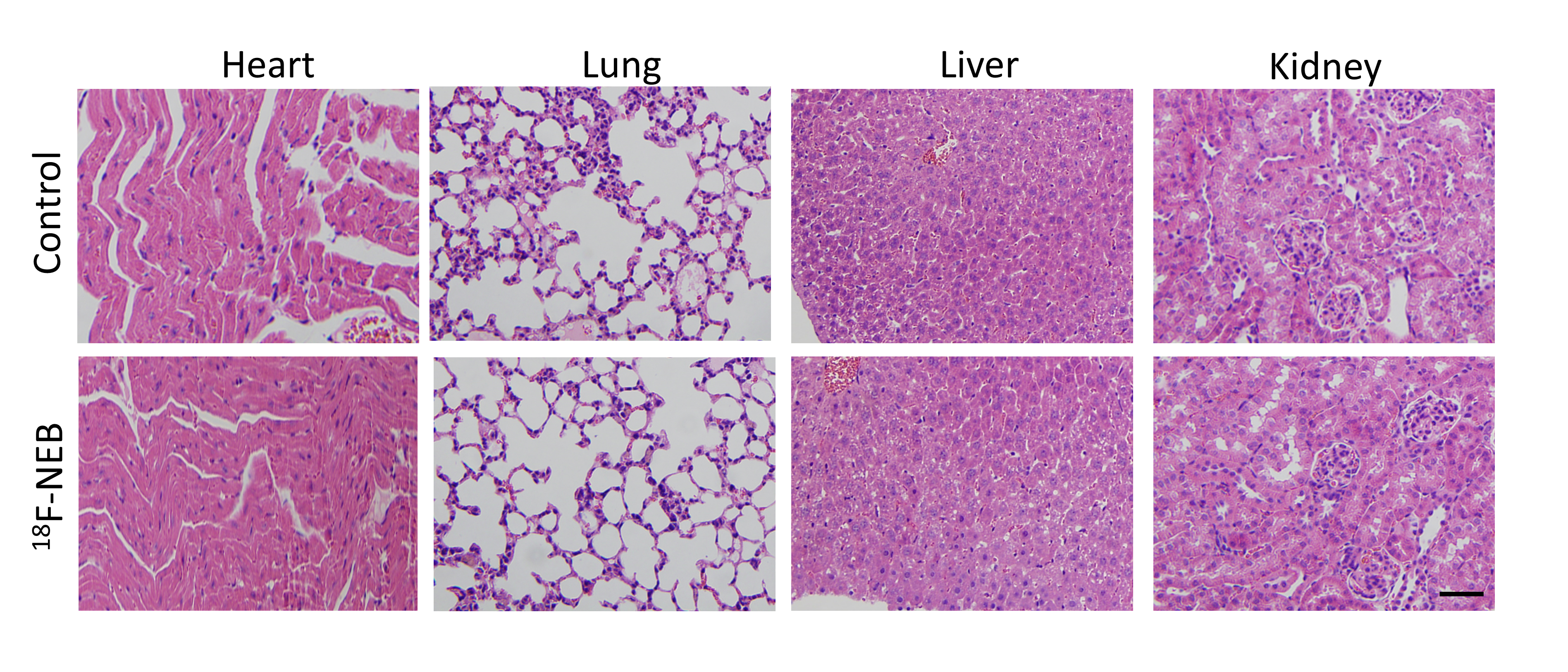

Supplement: Supplementary Figure S3 — H & E stained images of major organs (heart, lung, liver and kidney), harvested from mice 24 h after injection with/without 18F-NEB. [file Image_3.TIF]
